# Prevalence of Brick Tea-Type Fluorosis in the Tibet Autonomous Region

**DOI:** 10.2188/jea.JE20150037

**Published:** 2016-02-05

**Authors:** Zhipeng Fan, Yanhui Gao, Wei Wang, Hongqiang Gong, Min Guo, Shengcheng Zhao, Xuehui Liu, Bing Yu, Dianjun Sun

**Affiliations:** 1 Center for Endemic Disease Control, Chinese Center for Disease Control and Prevention, Harbin Medical University, Key Lab of Etiology and Epidemiology, Education Bureau of Heilongjiang Province & Ministry of Health, Harbin, China; 2 The Institute of Endemic Disease Control, Tibet Autonomous Region Center for Disease Control and Prevention, Lhasa, China; 3 The Institute of Prevention and Treatment on Endemic Disease of Hulunbuir City, Zhalantun, China

**Keywords:** brick tea-type fluorosis, skeletal fluorosis, dental fluorosis, altitude, Tibet

## Abstract

**Background:**

The prevalence of brick tea-type fluorosis is high in Tibet because of the habit of drinking brick tea in this region. Brick tea-type fluorosis has become an urgent public health problem in China.

**Methods:**

A cross-sectional survey was conducted to investigate prevalence of brick tea-type fluorosis in all districts of Tibet using a stratified cluster sampling method. Dental fluorosis in children aged 8–12 years and clinical skeletal fluorosis in adults were diagnosed according to the national criteria. A total of 423 children and 1320 adults participated in the study. Samples of drinking water, brick tea, brick tea infusion (or buttered tea), and urine were collected and measured for fluoride concentrations by the fluoride ion selective electrode method.

**Results:**

The fluoride level in all but one of the brick tea samples was above the national standard. The average daily fluoride intake from drinking brick tea in all seven districts in Tibet was much higher than the national standard. The prevalence of dental fluorosis was 33.57%, and the prevalence of clinical skeletal fluorosis was 46.06%. The average daily fluoride intake from drinking brick tea (*r* = 0.292, *P* < 0.05), urine fluoride concentrations in children (*r* = 0.134, *P* < 0.05), urine fluoride concentrations in adults (*r* = 0.162, *P* < 0.05), and altitude (*r* = 0.276, *P* < 0.05) were positively correlated with the prevalence of brick tea-type fluorosis. Herdsmen had the highest fluoride exposure and the most severe skeletal fluorosis.

**Conclusions:**

Brick tea-type fluorosis in Tibet is more serious than in other parts of China. The altitude and occupational factors are important risk factors for brick tea-type fluorosis.

## INTRODUCTION

Approximately 99% of all fluoride retained in the human body is found in mineralized tissues, mainly in bone but also in enamel and dentin.^1^ Moderate levels of fluoride help to increase bone mass and prevent dental caries. However, exposure to high levels of fluoride can cause dental fluorosis (DF)—an undesirable developmental defect of tooth enamel during amelogenesis—and skeletal fluorosis (SF)—a condition marked by osteosclerosis and ligament calcifications and often accompanied by osteoporosis, osteomalacia, or osteopenia.^2^^–^^4^

Tea leaves can store up to 98% of the fluoride present in the surrounding air and soil.^5^ Brick tea is made of older leaves and stalks than other types of tea. Therefore, the fluoride concentrations are higher than other types of tea. Brick tea-type fluorosis, an endemic fluorosis, is caused by consumption of fluoride-containing brick tea. People can be chronically exposed to high levels of fluoride from drinking brick tea and are at risk of brick tea-type fluorosis. Brick tea-type fluorosis is characterized by mild DF in children and severe SF in adults. It was discovered by epidemiologic surveys in the 1980s among the minorities in remote western and northern border districts in China.^6^

In Tibet, the production modes of Tibetans are mainly agriculture and animal husbandry. Drinking brick tea or buttered tea, which is made of brick tea and butter, is a lifestyle habit of the majority of Tibetans and can help them digest food and ingest minerals and vitamins but also leads to high levels of fluoride exposure.^7^ Furthermore, high altitude could promote fluoride absorption and movement in the intestine, causing fluoride to be readily retained in the bone and muscle.^8^^–^^10^ For Tibetans, high-altitude residence might increase the risk of brick tea-type fluorosis.

The prevalence of brick tea-type fluorosis in Tibet has become an urgent public health problem in China. However, most current research on the conditions associated with brick tea-type fluorosis in Tibet selected limited numbers of districts from Tibet and cannot adequately assess the prevalence of brick tea-type fluorosis in this region. In this study, we sampled from all districts of Tibet, including Lhasa, Ali, Nagqu, Shigatse, Shannan, Linzhi, and Changdu, to investigate environmental conditions for fluoride exposure, the prevalence and severity of brick tea-type fluorosis, and disease-associated risk factors in Tibetans.

## MATERIALS AND METHODS

### Selection of survey villages

We used a stratified random cluster sampling method for collecting samples. Specifically, a typical county was selected from each investigation district. The towns in each county were then stratified by the population size, and four towns (both urban and rural) were selected randomly. Finally, a village (of which there are several in each town) was randomly selected as a survey village from each town.

### Study population, questionnaire, and sample collection

DF conditions in children were investigated in the primary schools. Due to the local conditions in Tibet, there is only one primary school in each town and no primary school in villages of the town. Therefore, we selected the corresponding 8- to 12-year-old children belonging to the survey villages as the study population in the primary school. Baseline information of the 8- to 12-year-old children was collected, including age, gender, and nationality. DF was diagnosed according to national criteria (WS/T 208-2011). Urine samples were collected using a 15 mL centrifuge tube and stored at 4°C. Ultimately, we recruited 423 children and collected 416 urine samples.

Adults in the survey villages were investigated using a questionnaire, and SF was assessed and clinically diagnosed according to national criteria (WS/T 192-2008). The standard questionnaire was designed to obtain demographic information, including age, gender, nationality, income, source of drinking water, personal history of brick tea consumption, and the volume of brick tea consumed daily. We randomly selected at least 50 subjects from each survey village. We included subjects who were aged over 16 years and who had been drinking brick tea for several years. However, due to different religious beliefs, geographical conditions, and personal preferences, the number of subjects in some survey villages was less than 50. In order to ensure an adequate sample size, another nearby village was chosen as a complement when less than 50 subjects were recruited from the randomly selected village. Only three villages were investigated in Changdu due to the long distances between villages and severe weather conditions. In total, 1320 adults were recruited in 29 villages of seven districts, and 1227 brick tea infusion (or buttered tea) samples and 1287 urine samples were collected. In addition, 29 drinking water samples and 107 brick tea samples, which represent generalized drinking water and brick tea exposures, were also collected. All samples were collected with a 15 mL centrifuge tube and stored at 4°C.

### Brick tea consumption

We obtained information on brick tea consumption through three indices: the volume of brick tea consumed daily, the fluoride concentrations in brick tea infusion, and the average daily fluoride intake from drinking brick tea.

### Measurement of fluoride concentrations

Using the fluoride ion-selective electrode method (WS/T 89-2006), fluoride concentrations in drinking water, brick tea, brick tea infusion (or buttered tea), and urine samples were measured. The average daily fluoride intake from drinking brick tea was calculated using the following formula:

$$\begin{array}{l} \text{Average daily fluoride intake from drinking brick tea (mg)}\\ \;=\text{Fluoride concentration of brick tea infusion (mg/L)}\\ \;\times \text{volume of brick tea consumed daily (L)} \end{array}$$

### Statistical analysis

We mainly used non-parametric statistical analysis methods. The Kruskal-Wallis *H* test and Nemenyi test were used to compare the differences in fluoride levels among districts in Tibet. The Chi-square test was used to compare differences in the prevalence of brick tea-type fluorosis among those districts. Spearman’s rank correlation was used to analyze the correlations between the average daily fluoride intake from drinking brick tea, urine fluoride concentrations, or altitude and the prevalence of brick tea-type fluorosis. To examine whether or not occupational factors could influence the brick tea-type fluorosis, the adult participants were grouped into herdsmen, farmers, and other occupational groups (eg, workers, teachers, and students). The Kruskal-Wallis *H* test and Chi-square test were used to compare the differences in fluoride exposure and the severity of SF among different professional groups. The data were analyzed using SPSS version 19.0 (IBM Corporation, Armonk, NY, USA). *P* values less than 0.05 were considered statistically significant.

### Ethics statement

Following the principle of medical ethics, the investigation and sample collection were carried out after obtaining informed consent from the study subjects. This study was approved by the Ethics Review Board of Harbin Medical University.

## RESULTS

### Fluoride exposure in Tibet

#### Fluoride concentrations in drinking water

Every survey village had a centralized water supply. We obtained one sample from each of 29 villages. Among the 29 drinking water samples, only one sample (from Ali) had a fluoride concentration (2.01 mg/L) above the national drinking water standards for fluoride (≤1.2 mg/L, GB 5749-2006). The other 28 samples had fluoride concentrations between 0.03 mg/L and 0.96 mg/L, with a mean value of 0.23 mg/L.

#### Fluoride concentrations in brick tea

Fluoride concentrations in brick tea were between 96.53 mg/kg and 1085.70 mg/kg. Only one sample (collected from Shigatse, with a fluoride concentration of 96.53 mg/kg) met the national standard (≤300 mg/kg, GB 19965-2005). The remaining 106 brick tea samples had fluoride concentrations ranging from 348.34 to 1085.70 mg/kg, which were all above the national standard. The median fluoride level of the 106 samples was 732.81 mg/kg, which was more than twice the national standard.

#### Fluoride exposure from drinking brick tea

All three indices of brick tea consumption, including volume of brick tea consumed daily, fluoride concentrations in brick tea infusion, and the average daily fluoride intake from drinking brick tea, showed significant differences among the seven districts (Table 1). The median volume of brick tea consumed daily was 3.20 L (interquartile range [IQR], 2.40–6.00 L), and consumption was significantly higher in Ali, Nagqu, and Changdu compared with other districts (*P* < 0.05). The median fluoride concentration in brick tea infusion (including buttered tea) samples was 8.29 mg/L (IQR, 4.96–10.81 mg/L). The concentration of fluoride in brick tea infusions in Nagqu was significantly higher than in other areas (*P* < 0.05). The median value of average daily fluoride intake from drinking brick tea in Tibet was 24.73 mg (IQR, 12.16–41.57 mg), and the average intakes in all seven districts were above the national standard (≤3.5 mg, GB 17018-2011), with the highest intake in Nagqu (55.42 mg).

**Table 1.** Fluoride exposure from drinking brick tea

District	Volume of brick-tea consumed daily (L)		Fluoride concentrations in brick-tea infusion (mg/L)		Average daily fluoride intake from drinking brick-tea (mg)	
		
	*n*	Median (IQR)	*n*	Median (IQR)	*n*	Median (IQR)
Lhasa	115	3.20 (2.50–3.20)	112	8.30 (6.53–9.64)	112	26.56 (17.26–31.17)
Ali	107	6.00 (3.20–6.40)^a^	103	6.93 (3.45–11.69)	103	29.95 (17.41–58.56)
Nagqu	311	6.00 (3.20–6.50)^a^	296	10.92 (9.61–12.16)^a,b^	287	55.42 (33.60–77.83)^a,b^
Shigatse	124	3.20 (2.00–3.43)^b,c^	108	2.09 (1.04–7.82)^a,b,c^	108	6.77 (2.25–19.90)^a,b,c^
Shannan	321	2.40 (2.00–3.20)^a,b,c^	296	9.18 (6.52–10.33)^c,d^	295	21.48 (13.66–31.78)^b,c,d^
Linzhi	199	2.40 (1.50–3.20)^a,b,c^	182	5.81 (2.43–7.86)^a,b,c,e^	180	9.97 (3.78–21.96)^a,b,c,e^
Changdu	135	4.80 (3.20–6.40)^a,d,e,f^	130	4.96 (4.07–4.96)^a,b,c,e^	129	24.61 (14.11–31.75)^c,d,f^
Total	1312	3.20 (2.40–6.00)	1227	8.29 (4.96–10.81)	1214	24.73 (12.16–41.57)

IQR, interquartile range.

The Kruskal-Wallis *H* test and Nemenyi test were used to compare differences in fluoride exposure among districts.

^a^*P* < 0.05, Compared with Lhasa.

^b^*P* < 0.05, Compared with Ali.

^c^*P* < 0.05, Compared with Nagqu.

^d^*P* < 0.05, Compared with Shigatse.

^e^*P* < 0.05, Compared with Shannan.

^f^*P* < 0.05, Compared with Linzhi.

#### Urinary concentrations of fluoride in children and adults

Urinary fluoride concentrations are shown in Table 2. The geometric mean of urinary concentrations of fluoride among the 416 children in our sample was 0.77 mg/L, which was below the national standard (≤1.4 mg/L, WS/T256-2005). Urinary concentrations of fluoride in children from Changdu and Nagqu were significantly higher than in those from Shannan and Shigatse (*P* < 0.05). The geometric mean of urinary concentrations of fluoride in the 1287 adults in our sample was 1.62 mg/L, which was slightly above the national standard (≤1.6 mg/L, WS/T256-2005). The median level was 1.71 mg/L, and concentrations ranged from 0.13 mg/L to 13.04 mg/L. Urinary concentrations of fluoride in adults from Ali and Changdu were highest among the seven districts (*P* < 0.05).

**Table 2.** Urinary concentrations of fluoride in children and adults

District	Urinary fluoride concentrations in children (mg/L)				Urinary fluoride concentrations in adult (mg/L)			
	
	*n*	Geometric mean	Median	Range	*n*	Geometric mean	Median	Range
Lhasa	85	0.71	0.76	0.17–5.90	109	1.61	1.60	0.27–6.44
Ali*	—	—	—	—	105	3.21	3.19^a^	0.73–13.04
Nagqu	62	1.03	1.17^a^	0.23–3.15	309	1.82	1.86^b^	0.31–8.33
Shigatse	116	0.70	0.69^c^	0.15–2.04	122	0.94	1.02^a,b,c^	0.13–6.25
Shannan	107	0.65	0.67^c^	0.11–2.76	311	1.54	1.68^b,d^	0.24–6.97
Linzhi	13	0.75	0.73	0.24–2.35	195	1.05	1.11^a,b,c,e^	0.14–12.46
Changdu	33	1.33	1.26^a,d,e^	0.47–4.43	136	2.57	2.50^a,c,d,e,f^	0.56–10.66
Total	416	0.77	0.79	0.11–5.90	1287	1.62	1.71	0.13–13.04

Investigations were not done in children in Ali due to bad traffic conditions.

The Kruskal-Wallis *H* test and Nemenyi test were used to compare differences in urinary concentrations of fluoride in children and adults among districts.

^a^*P* < 0.05, Compared with Lhasa.

^b^*P* < 0.05, Compared with Ali.

^c^*P* < 0.05, Compared with Nagqu.

^d^*P* < 0.05, Compared with Shigatse.

^e^*P* < 0.05, Compared with Shannan.

^f^*P* < 0.05, Compared with Linzhi.

### Prevalence of brick tea-type fluorosis in all districts of Tibet

#### Prevalence of dental fluorosis among children

In this study, 423 children were examined for DF. The prevalence of DF for all districts in Tibet was 33.57%. There were significant differences in DF among different districts through the Chi-square test (χ^2^ = 20.901, *P* < 0.05). Specifically, significantly increased prevalences of DF (above 30%) among children were seen in Nagqu and Lhasa (Table 3).

**Table 3.** Prevalence of dental fluorosis among children aged 8–12 years

District	*n*	Normal	Questionable	DF					Prevalence (%)	DF index^a^	Prevalent strength^b^

				Very mild	Mild	Moderate	Severe	Total			
Lhasa	87	42	6	12	21	6	0	39	44.83	0.86	Mild
Nagqu	63	29	2	9	23	0	0	32	50.79	0.89	Mild
Shigatse	118	85	0	16	14	3	0	33	27.97	0.45	Edge
Shannan	109	53	28	16	9	2	1	28	25.69	0.53	Edge
Linzhi	13	9	1	3	0	0	0	3	23.08	0.27	Negative
Changdu	33	16	10	3	3	0	1	7	21.21	0.55	Edge
Total	423	234	47	59	70	11	2	142	33.57	0.62	Mild

DF, dental fluorosis.

^a^Dental fluorosis (DF) index is a dynamic index that indicates the prevalent strength of dental fluorosis in a district quantitatively. DF index = (number of questionable DF × 0.5 + number of very mild DF × 1 + number of mild DF × 2 + number of moderate DF × 3 + number of severe DF × 4)/total number of participants surveyed.

^b^Indicates the severity of this disease in the district.

#### Prevalence of skeletal fluorosis among adults

Among the 1320 adults examined, the prevalence of SF was 46.06%. Of the subjects with SF, the prevalence of degree II and III SF (with physiological dysfunction) was 28.79%. There were significant differences in SF in different districts through the Chi-square test (χ^2^ = 213.362, *P* < 0.05). The prevalences of SF in Nagqu and Ali were among the highest, followed by Lhasa and Changdu (Table 4).

**Table 4.** Skeletal fluorosis prevalence among adults

District	The Median Altitude (m)	Clinical diagnosis of SF				

		*n*	Number of SF cases (%)	I (%)	II (%)	III (%)
Lhasa	3658	115	68 (59.13)	25 (21.74)	43 (37.39)	0 (0)
Ali	4500	107	80 (74.77)	25 (23.36)	53 (49.53)	2 (1.87)
Nagqu	4500	315	215 (68.25)	84 (26.67)	127 (40.32)	4 (1.27)
Shigatse	4000	124	44 (35.48)	19 (15.32)	24 (19.35)	1 (0.81)
Shannan	3700	322	89 (27.64)	36 (11.18)	52 (16.15)	1 (0.31)
Linzhi	3100	201	40 (19.90)	18 (8.96)	22 (10.95)	0 (0)
Changdu	3500	136	72 (52.94)	21 (15.11)	47 (34.56)	4 (2.94)
Total	3851	1320	608 (46.06)	228 (17.27)	368 (27.88)	12 (0.91)

SF, skeletal fluorosis.

### Risk factors for brick tea-type fluorosis

There was a positive relationship between average daily fluoride intake from drinking brick tea and SF prevalence (*n* = 1214, *r* = 0.292, *P* < 0.05). Similarly, there was a positive relationship between urinary concentrations of fluoride and DF prevalence in children (*n* = 416, *r* = 0.134, *P* < 0.05). A positive relationship was also seen between urinary concentrations of fluoride and SF prevalence in adults (*n* = 1287, *r* = 0.162, *P* < 0.05). In addition, a significant association was seen between altitude and SF prevalence among adults (*n* = 1320, *r* = 0.276, *P* < 0.05). However, we did not find any statistically significant relationships between altitude and DF prevalence in children (*n* = 423, *r* = −0.019, *P* = 0.7).

Furthermore, statistically significant differences in the volume of brick tea consumed daily, fluoride concentrations in brick tea infusions, the average daily fluoride intake from drinking brick tea, and urinary fluoride concentrations were found in different occupational groups (Table 5). Both fluoride intake from drinking brick tea and urinary fluoride concentrations were significantly higher in herdsmen than in farmers and other groups (*P* < 0.05). Similarly, herdsmen had a higher prevalence of SF and higher stages of SF than the other two groups (*P* < 0.05).

**Table 5.** Fluoride exposure in different occupational groups and clinical diagnosis of skeletal fluorosis

Occupational groups	Volume of brick-tea consumed daily (L)		Fluoride concentrations in brick-tea infusion (mg/L)		Average daily fluoride intake from drinking brick-tea (mg)	
		
	*n*	Median (IQR)	*n*	Median (IQR)	*n*	Median (IQR)
Herdsmen	418	6.00 (3.20–6.43)	398	10.81 (8.62–12.16)	390	48.45 (26.58–74.83)
Farmers	884	3.20 (2.00–3.20)^a^	818	6.77 (4.04–9.55)^a^	814	19.13 (9.04–29.92)^a^
Others	10	1.95 (1.15–3.20)^a^	11	7.36 (2.09–9.99)^a^	10	8.21 (3.93–20.69)^a^
Total	1312	3.20 (2.40–6.00)	1227	8.29 (4.96–10.81)	1214	24.73 (12.16–41.57)

Occupational groups	Urinary fluoride concentrations (mg/L)				Clinical diagnosis of SF				
	
	*n*	Median	Geometric mean	Range	*n*	Number of SF cases (%)	I (%)	II (%)	III (%)
Herdsmen	413	2.10	2.11	0.31–13.04	421	295 (70.07)	108 (25.65)	181 (42.99)	6 (1.43)
Farmers	863	1.52^a^	1.44	0.13–12.46	888	310 (34.91)^a^	117 (13.18)	187 (21.06)	6 (0.68)
Others	11	1.49^a^	1.23	0.33–2.94	11	3 (27.27)^a^	3 (27.27)	0 (0)	0 (0)
Total	1287	1.71	1.62	0.13–13.04	1320	608 (46.06)	228 (17.27)	368 (27.88)	12 (0.91)

IQR, interquartile range; SF, skeletal fluorosis.

The Kruskal-Wallis *H* test and Chi-square test were used to compare differences in fluoride exposure and severity of skeletal fluorosis among different professional groups.

^a^*P* < 0.05, Compared with herdsmen.

## DISCUSSION

The tea plant in Tibet is known as the most fluoride-contaminated plant in the world.^11^ High concentrations of fluoride have been found in tea plant leaves and roots and may increase linearly with age.^12^^,^^13^ Brick tea is a product of compressed tea, which is mainly made of old tea leaves and stalks; therefore, fluoride concentrations in brick tea are generally higher than in other types of tea. Fluoride concentrations in tea from the main brick tea-producing districts have been found to range from 261.7 mg/kg to 875.8 mg/kg.^14^ Because of the customs, habits, and living environments of ethnic minorities who live in the Tibet autonomous region, Inner Mongolia autonomous region, Xinjiang autonomous region, Qinghai province, Gansu province, and Sichuan province, brick tea has become a regular part of the diet. Therefore, brick tea-type fluorosis is a major public health problem in the western part of China.

According to our study, fluoride concentrations in drinking water in Tibet were in the normal range; however, fluoride concentrations in brick tea exceeded the national standard dramatically. The median level of fluoride in brick tea in Tibet was 732.81 mg in the present study, which was higher than that reported in previous studies in Inner Mongolia, Xinjiang, and Ningxia.^15^^–^^18^ In addition, we found that the median volume of brick tea consumed by Tibetans was more than 3 L/day and that the average daily fluoride intake was up to 24.73 mg, which was seven times the national standard (≤3.5 mg).

Furthermore, there was a significant difference in fluoride exposure among districts. The median daily fluoride intake from drinking brick tea in Ali, Nagqu, Changdu, and Lhasa was much higher than that in Shigatse and Linzhi. Similarly, the geometric mean of urinary fluoride concentrations in adults in Ali, Nagqu, Changdu, and Lhasa was about twice that in Shigatse and Linzhi. The findings of our study suggest the most prevalent type of fluorosis in Tibet is brick tea-type fluorosis.^19^

In the present study, DF among 8- to 12-year-old children was mainly mild, which was consistent with the results of a previous epidemiological investigation.^20^ Most children who participated in this study were living in dormitories on campus. The children in the school did not have the habit of drinking brick tea, resulting in less exposure from the consumption of brick tea. This may explain the predominance of mild DF seen in our study.^21^

In 2009, the national prevalence of drinking water-type SF in China was reported to be 7.08%.^22^ However, our study showed that the prevalence of SF in Tibet was 46.06%. Moreover, another epidemiological survey of brick tea-type fluorosis conducted in eight provinces in China showed that the prevalence of SF in Tibet was the highest of any province in the study area (77.23%).^23^ Therefore, the prevalence of brick tea-type fluorosis in Tibet was higher than drinking water-type fluorosis nationwide, and Tibet was the area with the highest prevalence of brick tea fluorosis. The correlation of fluoride exposure from the consumption of brick tea with SF prevalence suggests that the consumption of brick tea containing high levels of fluoride was the etiological factor for SF in Tibet.^24^

There were significant differences in the prevalence of brick tea fluorosis among the investigated districts in Tibet. The prevalence of SF in Nagqu and Ali was higher than in Lhasa and Changdu, and Linzhi had the lowest prevalence. The average daily fluoride intake from drinking brick tea in Ali was not high, but the prevalence of brick tea-type fluorosis was the highest among all the districts, which suggests that some other factors may be involved in the brick tea-type fluorosis in Tibet. The average altitude of the investigated districts in Tibet was more than 3000 m. Among all of the districts studied, Ali and Nagqu have the highest altitude (4500 m), and Linzhi has the lowest altitude (3100 m). Our results showed a positive correlation between altitude and SF prevalence, which is consistent with previous reports.^4^^,^^25^ Some researchers have suggested that the alterations of acid-base balance in organisms caused by hypoxia at high altitude could decrease urinary excretion of fluoride and thus increase fluoride retention in the body.^26^ In addition, edema in liver and kidneys caused by hypoxia has been seen in people living at high altitude.^27^ Our previous animal experiments on brick tea-type fluorosis also suggested that epiphyseal cartilage damage, liver hydropic degeneration, and kidney coagulative necrosis were more severe in rats in the high-altitude group than in the low-altitude group.^28^

The present study found that the prevalence of brick tea-type fluorosis was significantly related to occupational factors. Higher prevalence was found in villages where herding was the predominate occupation, while lower prevalence was observed in the agriculture villages. Because of the tough living condition, the herdsmen drink brick tea for vitamins and minerals. This may be the reason why the SF prevalence among the herdsmen was higher.

This study was a systematic investigation of the prevalence of brick tea-type fluorosis in the Tibet autonomous region. Based on the characteristics of brick tea-type fluorosis in Tibet and the associated risk factors, we suspect that the etiological factor of brick tea-type fluorosis in Tibet was drinking brick tea that contained high concentrations of fluoride. Therefore, strengthening the supervision of brick tea production and ensuring the circulation of low-fluoride brick tea in the market could be effective measures to reduce the prevalence of brick tea-type fluorosis in the Tibet autonomous region.
